# Impact on Health Disparities: Secondary Analysis of an Unplanned Extubation Quality Improvement Study

**DOI:** 10.1097/pq9.0000000000000922

**Published:** 2026-09-22

**Authors:** Gayathri Sreenivasan, Parvathy Krishnan, Lance A. Parton, Meenakshi Singh

**Affiliations:** From the * Division of Newborn Medicine, Tufts Medicine Pediatrics, Regional Neonatal Intensive Care Unit, Maria Fareri Children’s Hospital at Westchester Medical Center, Valhalla, N.Y.; † Tufts University School of Medicine, Floating Hospital for Children at Tufts Medical Center, Mass.; ‡ Department of Pediatrics, Maria Fareri Children’s Hospital at Westchester Medical Center, Valhalla, N.Y.; § Department of Pediatrics, Boston Children’s Health Physicians, Valhalla, N.Y.

## Abstract

**Introduction:**

Quality improvement (QI) initiatives have reduced unplanned extubation (UE) rates in neonatal intensive care units, but few studies have examined the equity of these improvements. An explicit equity focus in QI can reveal disparities that may otherwise remain unrecognized. This secondary analysis of a previously published UE prevention QI initiative aimed to identify baseline disparities in UE rates and determine whether these disparities narrowed following the implementation of a standardized best-practice bundle (BPB).

**Methods:**

We reviewed 6 months of preintervention medical record data and stratified UE rates by race/ethnicity, preferred primary language, and socioeconomic status (insurance as a proxy). Using the Model for Improvement, the QI team implemented the BPB components sequentially and uniformly across the neonatal intensive care unit. Data collection continued throughout implementation. Statistical process control (U) charts were used to examine baseline disparities and assess changes over time.

**Results:**

At baseline, non-Hispanic Black infants, patients from English-speaking families, and Medicaid-insured patients had higher UE rates. Following the implementation of the BPB, UE rates decreased across all subgroups. Control charts showed special-cause variation beginning in December 2022. Differences in UE rates across racial/ethnic, language, and socioeconomic groups narrowed.

**Conclusions:**

UE rates were higher among racial minority and lower socioeconomic status populations at baseline. A standardized QI approach was associated with lower UE rates across all subgroups and reduced baseline disparities.

## INTRODUCTION

Despite advances in neonatal care, inequities in the quality and outcomes of care persist across neonatal intensive care unit (NICU) populations. Although neonatal care should ideally be delivered equitably to all infants, racial and ethnic disparities in both healthcare delivery and neonatal outcomes persist.^1–7^ Studies show that non-Hispanic (NH) Black and Hispanic infants are more likely to receive care in hospitals with lower quality ratings and experience higher rates of morbidity and mortality.^3–5^ Disparities are reported in complications of prematurity, including necrotizing enterocolitis, retinopathy of prematurity, and intraventricular hemorrhage.^6^ Furthermore, systematic reviews have highlighted racial and ethnic inequities in NICU care, particularly among Black infants, and significant variation in the quality of care between and within NICUs.^2,7^ Together, these findings suggest persistent disparities affecting neonates across the continuum of care, beginning before birth and extending throughout the neonatal period.^8^

It is imperative to identify and understand these disparities in perinatal and neonatal care to address them and provide equitable care.^9,10^ Although disparities have been reported in neonatal outcomes, including morbidity and mortality, there is no specific study evaluating disparities in unplanned extubation (UE) rates. This event could indirectly influence overall outcomes. Multiple studies have focused on strategies to reduce UE in the NICU population, but few have explored whether disparities exist in UE events or in response to UE reduction strategies in this vulnerable population. ^7,11–13,14^Given the well-documented racial, ethnic, and socioeconomic disparities across a range of neonatal outcomes, it is important to determine whether similar inequities exist in UE events and in the effectiveness of UE reduction strategies. In healthcare quality improvement (QI), equality refers to the provision of identical care to all patients. In contrast, equity emphasizes tailoring interventions to address differing needs and structural barriers to achieve fair and just outcomes.^15^

A Specific, Measurable, Action-oriented, Relevant, Time-bound, Inclusive and Equitable (SMARTIE) aim builds upon the traditional Specific, Measurable, Action-oriented, Relevant, and Time-bound (SMART) framework commonly used in QI. The SMARTIE approach intentionally adds inclusion and equity.^16–18^ By doing so, it emphasizes that improvement efforts should not only achieve measurable outcomes but also address inequities and ensure that progress benefits all patient populations fairly. Our prior QI initiative was guided by a SMART aim to reduce the overall UE rate by at least 10% by December 2022 through the implementation of a standardized best-practice bundle (BPB) across the NICU.^19^ In this secondary analysis, we extended the evaluation to the inclusion and equity components of the SMARTIE framework by stratifying UE rates by race/ethnicity, preferred primary language, and socioeconomic status (SES) to assess differences across patient groups.

## METHODS

The study was conducted in a 58-bed level IV regional referral NICU caring for a diverse patient population. The unit is an open-bay NICU and provides comprehensive medical and surgical care for critically ill neonates. A multidisciplinary team provides care. The team includes neonatologists, fellows, nurse practitioners (NPs), residents, respiratory therapists (RTs), and bedside nurses. The unit typically maintains a 1:2 nurse-to-patient ratio and provides individualized staffing for higher acuity infants as needed. The clinical team manages mechanically ventilated infants using conventional ventilation as well as advanced modes of respiratory support, including high-frequency oscillatory ventilation, high-frequency jet ventilation, and volumetric diffusive respiration. A substantial proportion of infants require invasive mechanical ventilation during the early neonatal period, making endotracheal tube (ETT) management and securement critical components of patient safety. Baseline review demonstrated higher-than-expected rates of UE, prompting focused QI efforts.

**Fig. 1.**
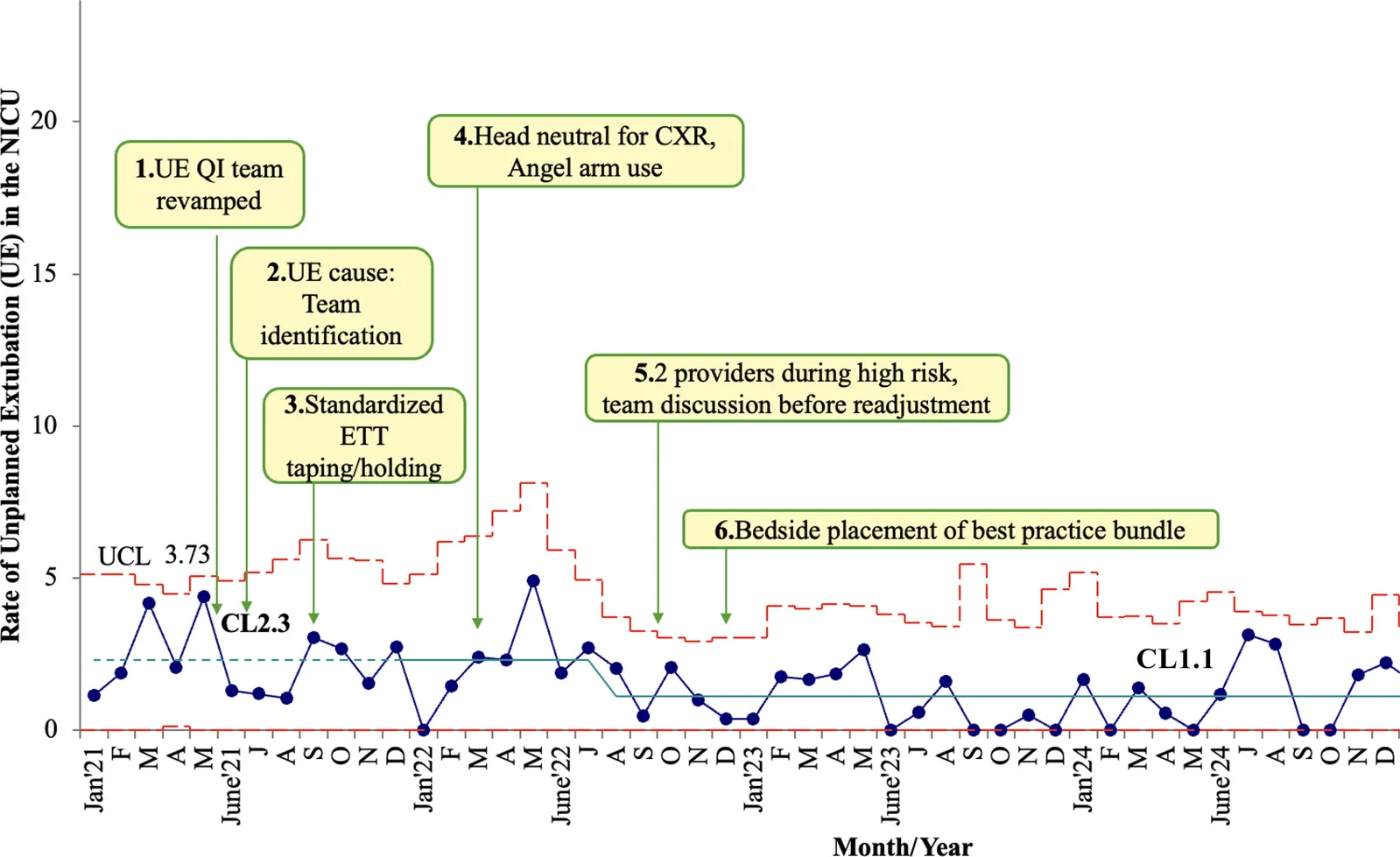
Statistical process control U chart of UE rates per 100 ventilator days over the study period. Arrows indicate the timing of sequential BPB implementation. CXR, chest X-ray.

**Fig. 2.**
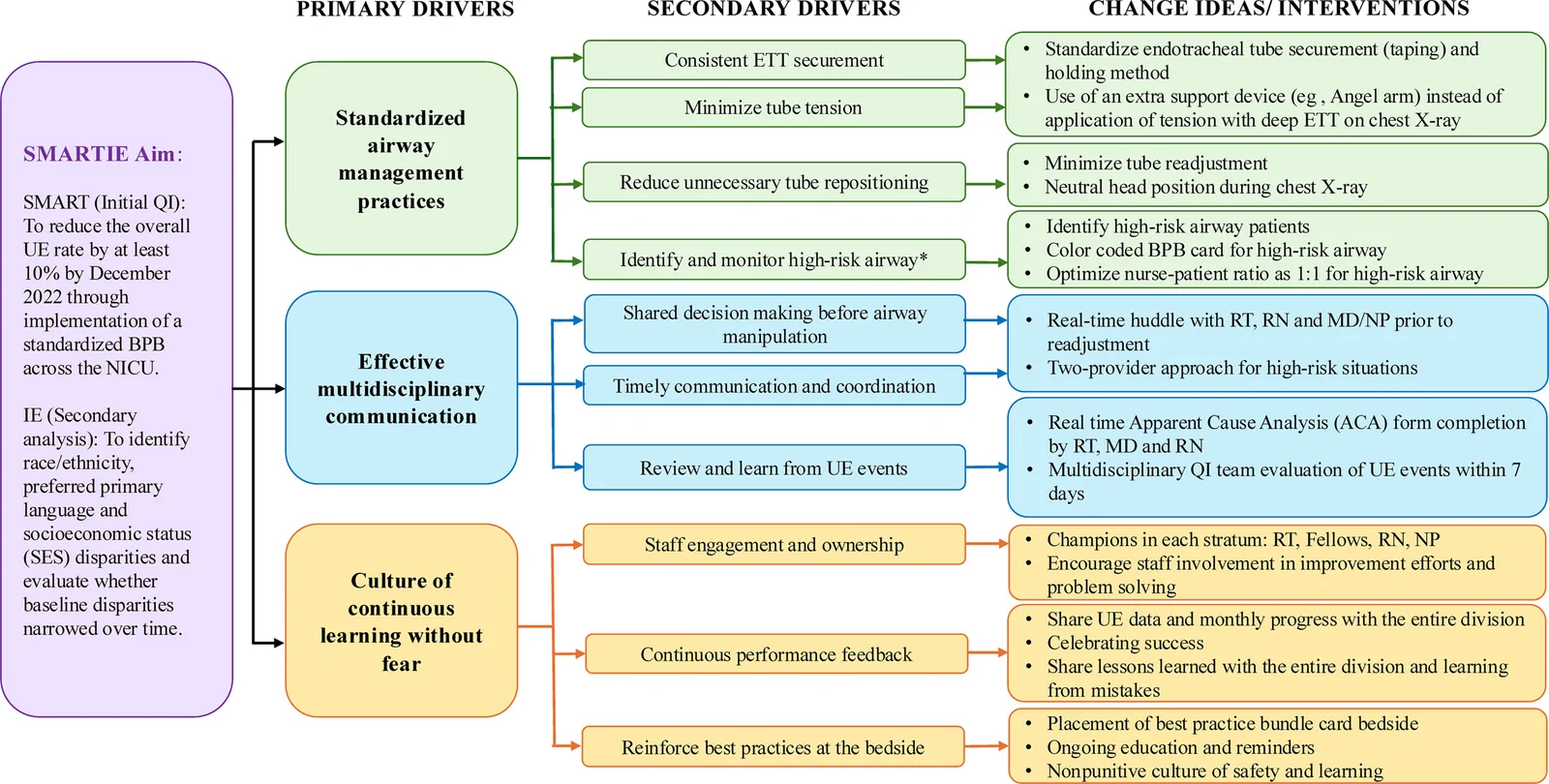
Key driver diagram developed to guide the implementation of standardized, unit-wide interventions aimed at reducing UEs in the NICU. *High-risk airways: difficult airway, weight<600g, previous UE.

We established a multidisciplinary UE QI team comprising neonatologists, fellows, NPs, RTs, and bedside nurses. Pareto chart analysis identified the most common contributors to UE events. Following each event, the involved doctor of medicine (MD)/registered nurse (NP), RT, and RN held a multidisciplinary discussion. They completed an apparent cause analysis form in real time to identify contributing factors and guide subsequent improvement efforts. The QI team implemented the BPB for intubation care in a stepwise manner. The team introduced each component after achieving adequate uptake of the preceding intervention. The BPB included (1) standardization of ETT securement methods, including holding and taping (September 2021); (2) use of an angel-arm device to reduce tension on the ETT and minimize unnecessary tube readjustment by evaluating tube position with a neutral midline head position during chest radiography (March 2022); (3) multidisciplinary huddles involving the RT, RN, and MD/NP before ETT adjustment and the presence of 2 providers during high-risk procedures (October 2022); and (4) placement of a bedside BPB reminder card (December 2022) (Fig. 1). A key driver diagram guided the BPB interventions (Fig. 2). The QI team implemented the comprehensive BPB by December 2022 and applied it uniformly across all patient groups.

Baseline UE data collection included 6 months of preintervention data (January 2021–June 2021). Data collection continued through intervention implementation and the postintervention period. The primary outcome was the UE rate, defined as the number of UE events per 100 ventilator days. Stratified analyses assessed baseline disparities in UE across racial/ethnic, preferred primary language, and SES groups. We also evaluated changes in these disparities over time following BPB implementation.

We used statistical process control methods to distinguish common-cause from special-cause variation (SCV) and evaluate temporal associations between changes in UE rates and BPB implementation. We analyzed the data using Microsoft Excel and QI Macros. Overall and stratified control “U” charts displayed UE rates over time. Eight or more consecutive points above or below the centerline constituted a shift, indicating SCV and prompting the establishment of a new centerline. Points outside the control limits also indicated SCV.^16^ This report follows the Standards for QUality Improvement Reporting Excellence 2.0 guidelines.^20^

### Ethical Considerations

This project was reviewed by the institutional review board and deemed exempt as a QI initiative. No patient consent was required, and all data were de-identified before analysis.

## RESULTS

### Baseline Data

Baseline UE data were collected from January 2021 to June 2021. Monthly UE events ranged from 0 to 14, and total ventilator days ranged from 61 to 437 days per month. The UE rate was defined as the number of UE events per 100 ventilator days. Baseline data were stratified based on race/ethnicity, preferred primary language, and SES.

**Fig. 3.**
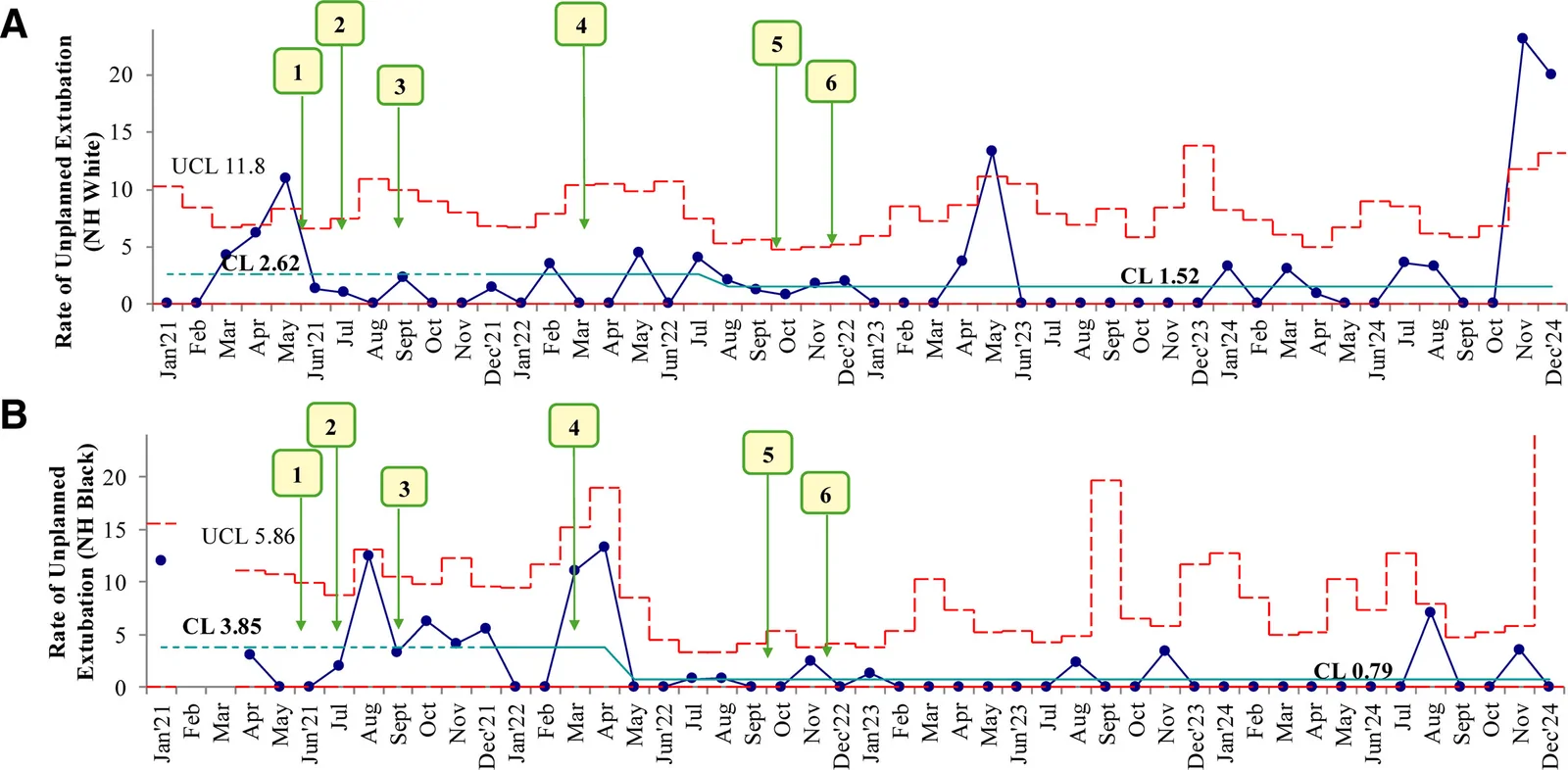
Statistical process control U chart of UE rates per 100 ventilator days among NH White infants (A) and NH Black infants (B). Arrows indicate the timing of sequential BPB implementation. CL, central line; UCL, upper control limit.

**Fig. 4.**
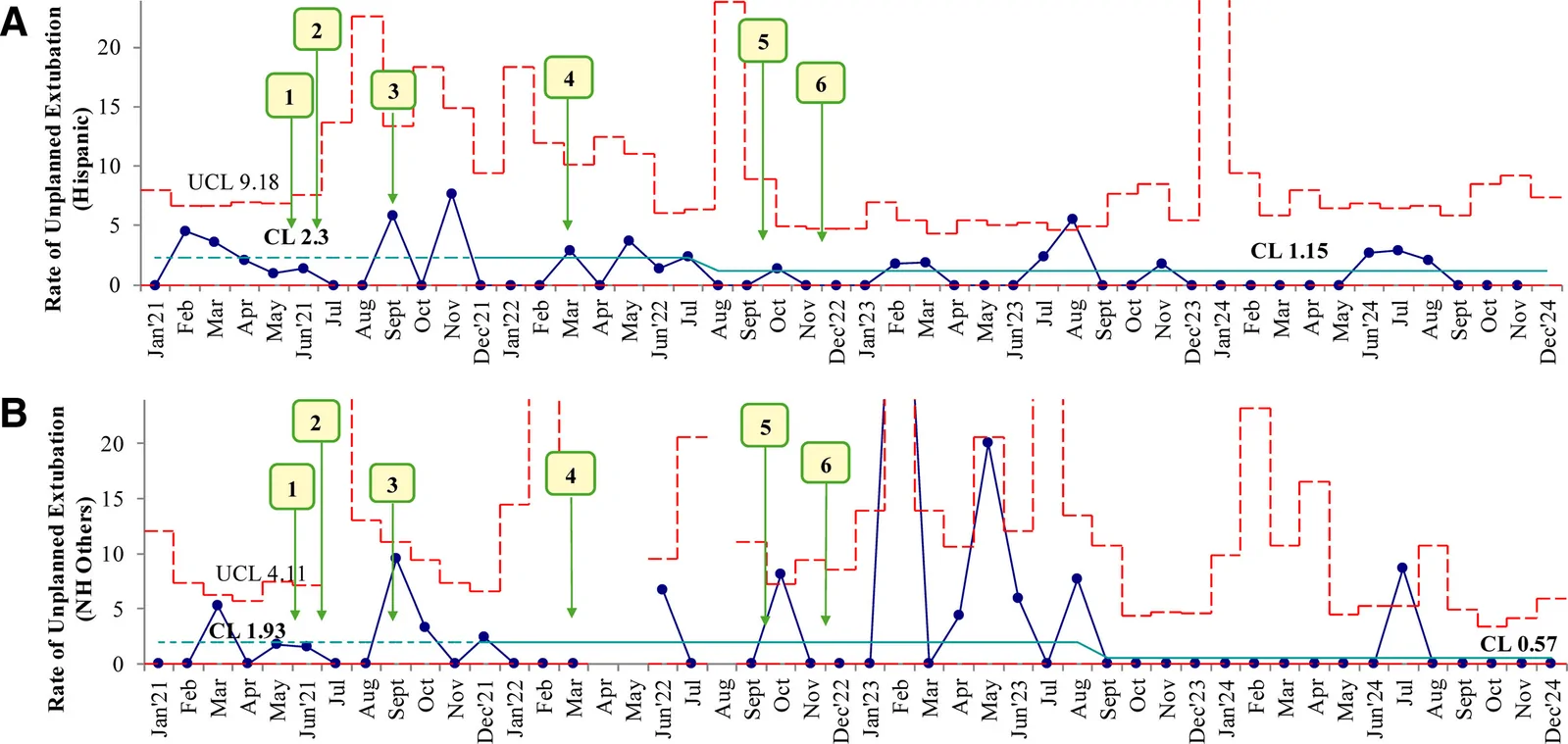
Statistical process control U chart of UE rates per 100 ventilator days among Hispanic infants (A) and NH other race/ethnicity infants (B). Arrows indicate the timing of sequential BPB implementation.

At baseline, NH Black infants, patients from English-speaking families, and Medicaid-insured patients had higher UE rates. NH Black infants had the highest mean UE rate (3.85), followed by NH White (2.62), Hispanic (2.30), and NH other race/ethnicity groups (1.93) (Figs. 3, 4). Hispanic infants comprised the largest proportion of NICU admissions over time across racial/ethnic groups.

**Fig. 5.**
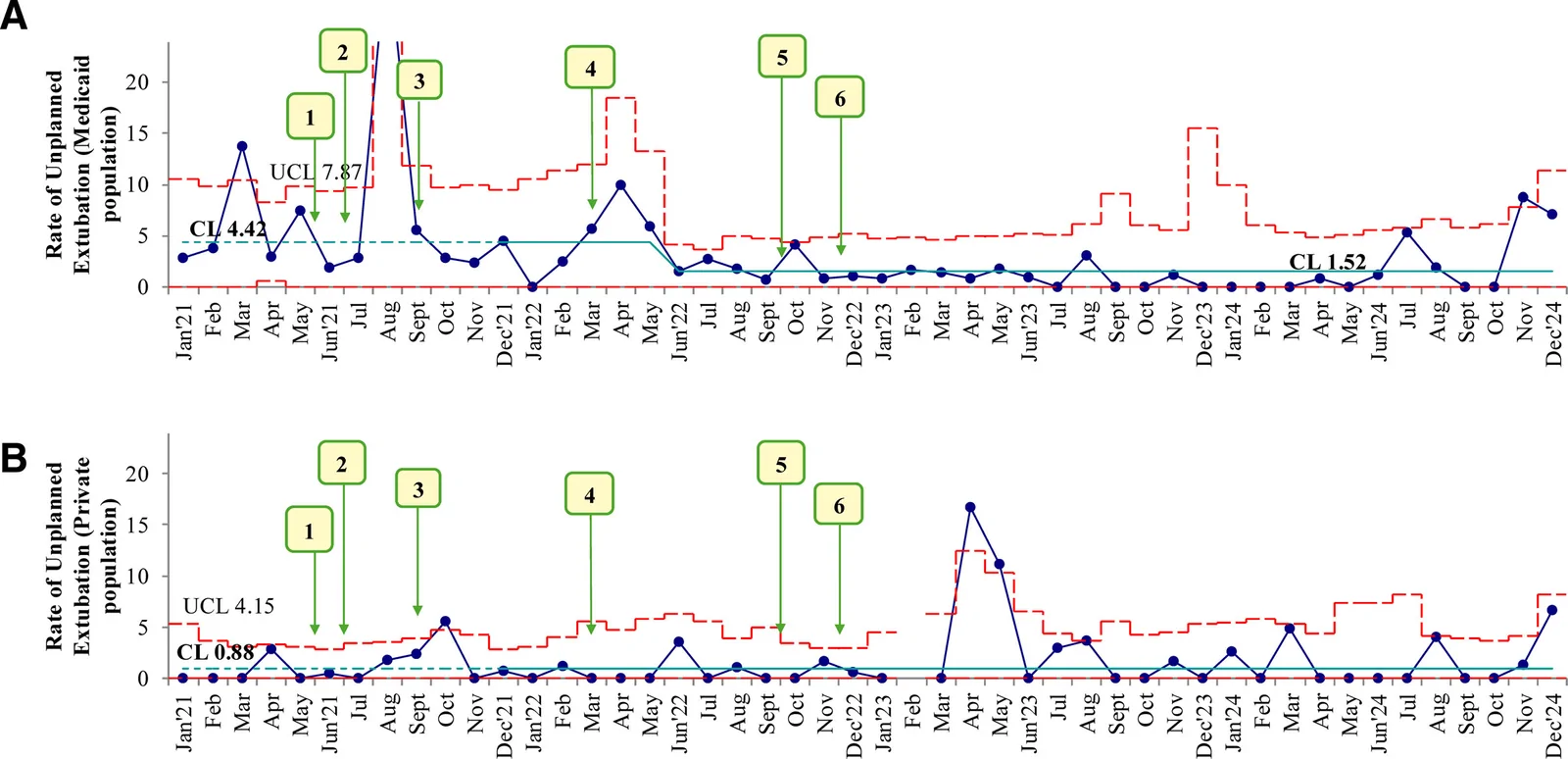
Statistical process control U chart of UE rates per 100 ventilator days among Medicaid-insured infants (A) and privately insured infants (B). Arrows indicate the timing of sequential BPB implementation.

**Fig. 6.**
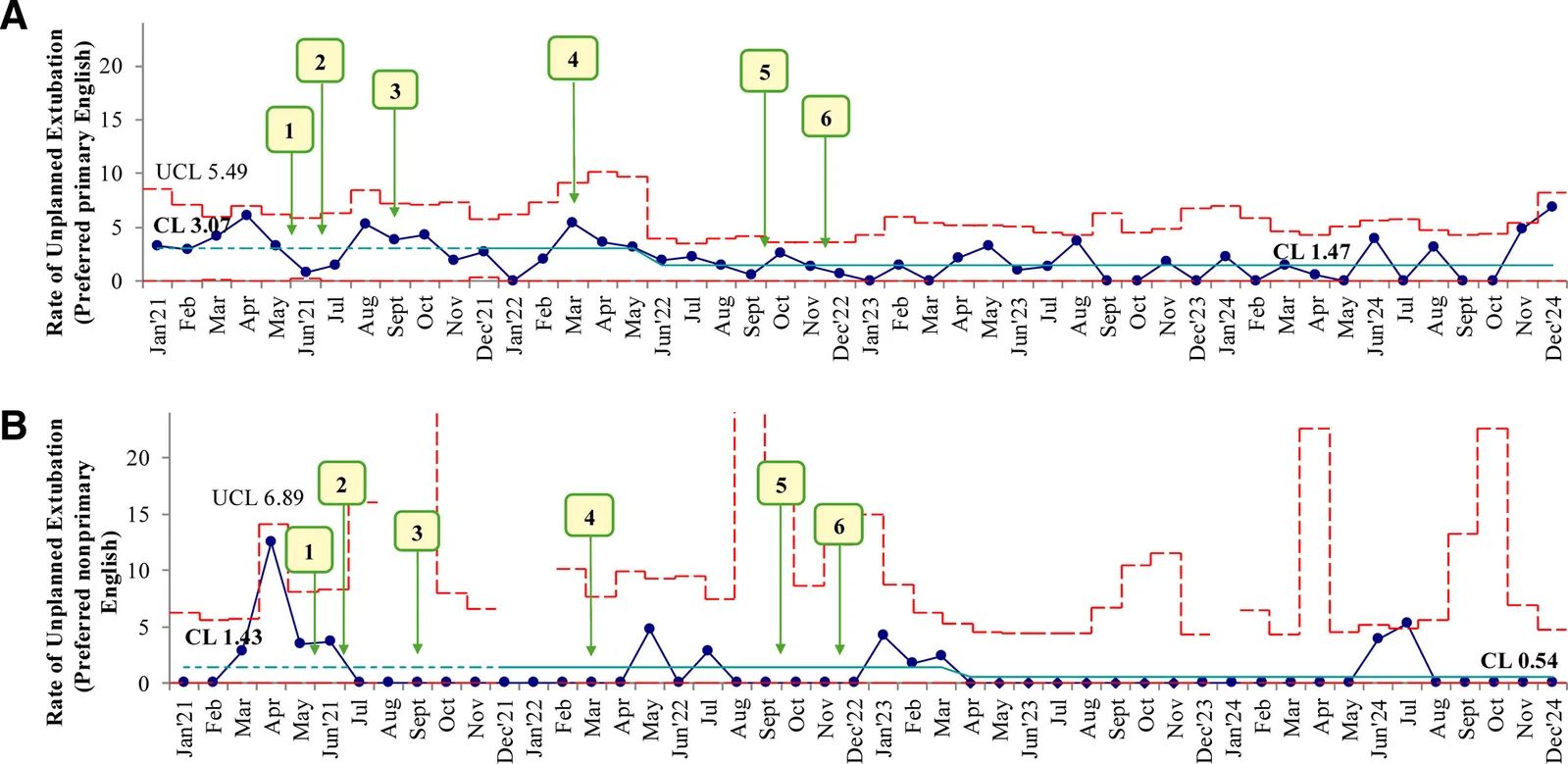
Statistical process control U chart of UE rates per 100 ventilator days among preferred primary English-speaking families (A) and non–primary English-speaking families (B). Arrows indicate the timing of sequential BPB implementation.

Stratified admission data showed that Medicaid-insured infants and those from preferred primary English-speaking families comprised most admissions. Within this group, Medicaid-insured patients had higher UE rates than privately insured patients (mean 4.42 versus 0.88) (Fig. 5). Infants from English-speaking families also had higher UE rates compared with those from non–primary English-speaking families (mean 3.07 versus 1.43) (Fig. 6).

### Outcome

Following the implementation of the BPB, UE rates across racial/ethnic groups, including NH Black, NH White, Hispanic, and NH other race/ethnicity groups, demonstrated SCV, with control charts meeting criteria for a centerline shift.^17^ UE rates decreased from 3.85 to 0.79 events per 100 ventilator days among NH Black infants (79.5% reduction), from 2.62 to 1.52 among NH White infants (42% reduction), from 2.30 to 1.15 among Hispanic infants (50% reduction), and from 1.93 to 0.57 among NH infants of other race/ethnicity (70.5% reduction).

By preferred primary language, both English-speaking and non–English-speaking families demonstrated reductions in UE rates with SCV and qualifying shift changes on control charts. UE rates decreased from 3.07 to 1.47 events per 100 ventilator days among preferred primary English-speaking families (52.1% reduction) and from 1.43 to 0.54 among preferred non–primary English-speaking families (62.2% reduction).

By SES, Medicaid-insured patients demonstrated reductions in UE rates with SCV and a qualifying shift change, with rates decreasing from 4.42 to 1.52 events per 100 ventilator days (65.6% reduction), whereas the UE rate among privately insured patients remained low at 0.88 events per 100 ventilator days without a centerline shift. Overall, the differences in UE rates among racial/ethnic, language, and SES groups narrowed following the implementation of the BPB. Occasionally, data points crossed the upper control limit in multiple subgroups, meeting criteria for SCV in an undesirable direction.^17^ Review of these periods demonstrated lower total ventilator days in the corresponding months, which were temporally associated with higher UE rates. As these changes were random and nonsustained, the team decided not to modify the centerline.

## DISCUSSION

Racial/ethnic, language, and socioeconomic disparities were evident in UE events within our NICU population. These disparities narrowed following the implementation of standardized care interventions. This secondary analysis extends our previously published QI study, which demonstrated a 48% reduction in the overall UE rate following the stepwise implementation of the BPB.^19^ This project provides a novel evaluation of UE outcomes through a health disparities lens by examining improvements across racial/ethnic, linguistic, and socioeconomic groups. The BPB was implemented uniformly across all mechanically ventilated infants, and outcomes were subsequently evaluated across demographic groups to determine whether baseline differences narrowed following implementation.

Our findings demonstrated that NH Black infants, Medicaid-insured (a proxy for lower SES), and infants whose maternal primary language was English experienced disproportionately higher baseline rates of UE events. The higher baseline UE rates among NH Black and Medicaid-insured infants align with previously reported disparities in neonatal outcomes and NICU care delivery.^3,5–7,13,21–23^ Our findings similarly indicate that lower SES populations may be at elevated risk for UE events and related complications. In the outpatient setting, disparities may result from inequities in resources, parental engagement, and care coordination.^24,25^ In the inpatient setting, systemic implicit biases may compound these gaps.^26,27^

Families with English as their primary language had higher UE rates. This finding was unexpected, as prior literature often describes disparities affecting non–English-speaking families.^28,29^ One possible explanation is heightened institutional attention to communication quality and protocol adherence for non–English-speaking families, which may have mitigated UE risk in this group. This interpretation aligns with qualitative findings suggesting that proactive, culturally responsive communication strategies can reduce disparities in care delivery.^2^ Notably, although Hispanic infants comprised the largest proportion of our cohort, they accounted for relatively few UE events. This pattern may reflect the overlap between Hispanic ethnicity and a preferred non–English language. All families in the non–English language group identified Spanish as their primary preferred language.

Following implementation of standardized interventions, UE rates declined, and disparities narrowed across racial/ethnic, language, and SES categories. These findings suggest that standardized, team-based protocols may reduce variability in care processes and improve outcomes across diverse patient populations.^8,11,30^ Similar QI initiatives have shown that broadly implemented safety interventions can benefit multiple patient subgroups and may contribute to reductions in observed disparities.^7,10,30,31^

Importantly, our interventions did not target the underlying causes of disparities. Rather, the bundle comprised standardized evidence-based practices that the team applied universally to all mechanically ventilated infants. Although UE rates decreased across demographic groups, our findings do not explain the reasons for baseline disparities or establish why some groups experienced greater improvements than others. Persistent differences, particularly by insurance status, highlight the need for future studies using equity-focused frameworks. Such studies should identify factors contributing to inequitable outcomes and evaluate targeted strategies.

Several factors supported sustained improvement: strong institutional leadership, monthly multidisciplinary QI meetings, ongoing identification and correction of BPB deviations, and continued frontline staff enthusiasm and engagement. A nonpunitive culture emphasized shared learning from UE events and dissemination of lessons learned. This approach fostered open communication and encouraged continued adherence to prevention strategies. The integration of key bundle components into daily clinical workflows helped maintain awareness of UE prevention practices and sustain improvements over time.

Several challenges to long-term sustainability remain. Ongoing reinforcement and repeated education are necessary because of staff turnover, rotating trainees, and evolving clinical practice environments. Sustained success also depends on continued multidisciplinary engagement and monitoring of adherence to bundle elements. In addition, variation in UE definitions and reporting across institutions may present challenges for benchmarking and comparison of outcomes. Future improvement opportunities include increasing skin-to-skin care among mechanically ventilated infants, developing standardized extubation readiness guidelines, and reducing variability in ventilator weaning practices. Current weaning practices remain largely provider-dependent. Continued attention to these areas may further strengthen patient safety efforts and support sustained reductions in UE rates.

Overall, this project contributes to growing evidence that health equity is essential to providing high-quality care. Integrating equity considerations into QI design and implementation may help ensure that improvements extend beyond aggregate outcomes to benefit all patients across diverse racial, linguistic, and socioeconomic backgrounds.

## STUDY LIMITATIONS

This secondary analysis relied on self-reported race/ethnicity and preferred primary language. When infant-level demographic data were missing, information was supplemented from the maternal record; however, this approach did not capture paternal contributions. The dataset lacked baseline NICU admission data stratified by preferred language. Therefore, language comparisons included only intubated infants and may not fully reflect the language distribution of the overall NICU population.

Baseline data availability was limited, as the intervention had already been implemented before detailed baseline data collection, restricting our ability to fully characterize preintervention performance. Although this project identified disparities in UE rates, it did not examine the underlying mechanisms contributing to these differences. We monitored process measures at the unit level but did not stratify them by race/ethnicity, language, or SES; therefore, we were unable to evaluate whether adherence to individual bundle components differed across demographic groups. Balancing measures were not prospectively collected, limiting our ability to assess potential unintended consequences of the intervention. Given that disparities narrowed following the implementation of standardized care, equity-specific interventions were not pursued, precluding the prospective application of an equity-focused framework. Finally, equity-focused frameworks are most effective when applied prospectively at the initiation of QI projects, allowing disparity identification in real time rather than retrospectively. Future QI initiatives should consider incorporating stratified process measures, balancing measures, and prospective equity-focused interventions to better evaluate equitable implementation and unintended consequences across patient populations.

## CONCLUDING SUMMARY

This secondary analysis of QI project data identified disparities in UE rates across racial/ethnic, language, and SES groups within a NICU. Implementation of standardized care processes was associated with reductions in overall UE rates and narrowing of these disparities. These findings highlight the potential for standardized processes to advance equity. They also underscore the importance of intentionally incorporating equity considerations prospectively into QI design and implementation. QI initiatives can help ensure that safety improvements extend beyond aggregate outcomes to benefit all patient populations equitably.

## ACKNOWLEDGMENTS

The authors acknowledge the cooperation and support of the UE Prevention Team members: Diana D’Agrosa, Alexandra Pickard, DeBorah Briston, and Susan R. Malfa, as well as the NICU nursing staff, RTs, and medical staff at the study hospital. During the preparation of this work, the author(s) used generative AI (ChatGPT, OpenAI) to assist with language editing, improving clarity, and refining the phrasing of the article. After using this tool/service, the author(s) reviewed and edited the content as needed and take full responsibility for the content of the publication.
